# SnoRNA *Snord116 (Pwcr1/MBII-85*) Deletion Causes Growth Deficiency and Hyperphagia in Mice

**DOI:** 10.1371/journal.pone.0001709

**Published:** 2008-03-05

**Authors:** Feng Ding, Hong Hua Li, Shengwen Zhang, Nicola M. Solomon, Sally A. Camper, Pinchas Cohen, Uta Francke

**Affiliations:** 1 Department of Genetics, Stanford University, Stanford, California, United States of America; 2 Department of Psychiatry and Behavioral Science, Stanford University, Stanford, California, United States of America; 3 Department of Human Genetics, University of Michigan, Ann Arbor, Michigan, United States of America; 4 Department of Pediatrics, University of California Los Angeles, Los Angeles, California, United States of America; 5 Department of Pediatrics, Stanford University, Stanford, California, United States of America; University of Massachusetts Medical School, United States of America

## Abstract

Prader-Willi syndrome (PWS) is the leading genetic cause of obesity. After initial severe hypotonia, PWS children become hyperphagic and morbidly obese, if intake is not restricted. Short stature with abnormal growth hormone secretion, hypogonadism, cognitive impairment, anxiety and behavior problems are other features. PWS is caused by lack of expression of imprinted genes in a ∼4 mb region of chromosome band 15q11.2. Our previous translocation studies predicted a major role for the C/D box small nucleolar RNA cluster *SNORD116 (PWCR1/HBII-85*) in PWS. To test this hypothesis, we created a ∼150 kb deletion of the >40 copies of *Snord116 (Pwcr1/MBII-85)* in C57BL/6 mice. *Snord116del* mice with paternally derived deletion lack expression of this snoRNA. They have early-onset postnatal growth deficiency, but normal fertility and lifespan. While pituitary structure and somatotrophs are normal, liver *Igf1* mRNA is decreased. In cognitive and behavior tests, *Snord116del* mice are deficient in motor learning and have increased anxiety. Around three months of age, they develop hyperphagia, but stay lean on regular and high-fat diet. On reduced caloric intake, *Snord116del* mice maintain their weight better than wild-type littermates, excluding increased energy requirement as a cause of hyperphagia. Normal compensatory feeding after fasting, and ability to maintain body temperature in the cold indicate normal energy homeostasis regulation. Metabolic chamber studies reveal that *Snord116del* mice maintain energy homeostasis by altered fuel usage. Prolonged mealtime and increased circulating ghrelin indicate a defect in meal termination mechanism. *Snord116del* mice, the first snoRNA deletion animal model, reveal a novel role for a non-coding RNA in growth and feeding regulation.

## Introduction

As the leading genetic cause of obesity with a prevalence of 1 in 10,000 to 20,000, Prader-Willi syndrome (PWS) is a complex neuro developmental disorder characterized by neonatal hypotonia and failure to thrive, developmental delay, cognitive defects, hypogonadotropic hypogonadism, characteristic facial features, short stature, and behavioral abnormalities [1]. The most prominent feature of PWS is hyperphagia with an age of onset between 2 and 6 years, which if uncontrolled, can lead to morbid obesity and associated ailments that include type 2 diabetes, cardiovascular disease and early death [2]. The underlying mechanism of the hyperphagic behavior is unclear. PWS is caused by the inactivation or deletion of several imprinted, paternally expressed genes within 4 Mb of chromosome band 15q11.2 [3]. Molecular mechanisms include large deletions, maternal uniparental disomy for chromosome 15 or mutations involving the imprinting center. The current challenge in PWS research is to elucidate the contributions of individual loci to the genetic and molecular pathways that are affected, leading to PWS-associated phenotypes. Prior studies of spontaneous reciprocal translocations and deletions allowed us to narrow down the critical region within the common deletion interval to ∼121 kb [4], [5]. Within this interval resides a novel paternally expressed imprinted gene cluster, SNORD116 (the current approved gene symbol for *PWCR1/HBII-85* in human, GeneID: 692236, and *Pwcr1/MBII-85* in mouse, GeneID: 64243) that encodes multiple copies of a small nucleolar RNA (snoRNA) with short conserved sequence elements, called C and D boxes [6]–[8]. *SNORD116* in human, and *Snord116* in mouse, were the first mammalian snoRNA genes discovered to show uniparental (imprinted) expression. There are 27 copies of *SNORD116* in humans and more than 40 copies of *Snord116* in mice. These snoRNAs are located in introns of a “host gene” that is made up of non-coding (in human) exons of the large *SNRPN* transcript. Individual snoRNA molecules are generated during the pre-mRNA splicing process [9]. While the 96 bp snoRNA sequences are largely conserved between humans and mice, the host gene exons are not [6], [8]. The mouse build 37 genome database lists 41 copies, 26 of which have been mapped in regular intervals to three contigs on mouse chromosome band 7C, and the other 15 are not mapped, indicating that the genomic sequence in this region is still incomplete.

SnoRNAs of the C/D box class are found in all organisms and usually serve to direct the site-specific methylation of the ribose 2′-hydroxyl group of specific nucleotides in ribosomal RNA (rRNA) or small nuclear RNA (snRNA) by base-paring with the target sequences [10]. *SNORD116* belong to a novel class of snoRNAs, expressed most highly in brain in humans and exclusively in brain in mouse. Their putative target recognition sites are not complementary to any known rRNA, tRNA or snRNA sequences, and their biological targets are unknown.

**Figure 1:**
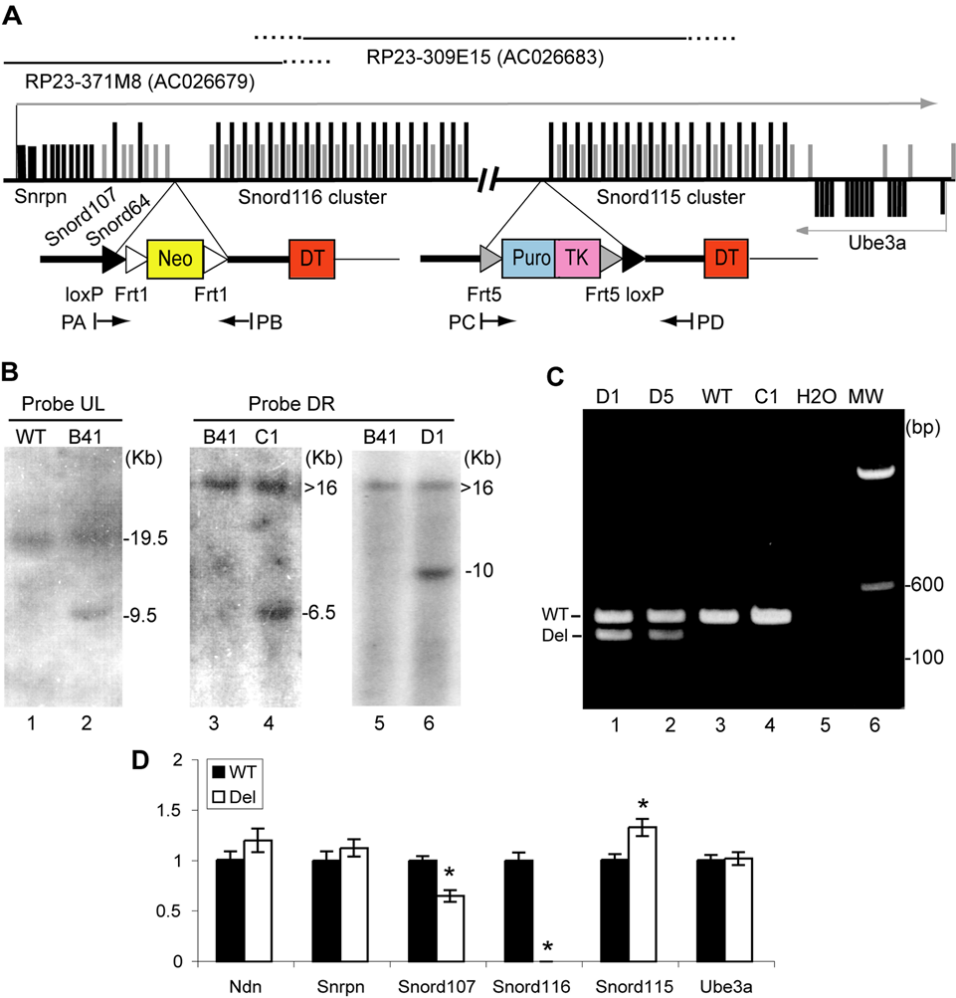
Derivation of Snord116 (Pwcr1/MBII-85) deletion mice. A. Genomic region and structure of targeting constructs (not to scale). Top: Location of BACs used for making constructs. Middle: Map of Prader-Willi syndrome critical region (Pwcr) on mouse chromosome 7C. Gray thin line with arrowhead, large transcript originating from the Snrpn promoter; short black bars, coding exons for Snrpn on the left, and for Ube3a on the right; short gray bars, non-coding exons; high black bars, snoRNAs (not representing accurate numbers of elements in clusters, there are at least 41 copies of Snord116). Bottom: Left, upstream construct; right, downstream construct. Thick lines represent mouse genomic sequence, and thin lines vector sequence. Black arrowheads, loxP sites; white arrowheads, Frt1 sites; grey arrowheads, Frt 5 sites. Neo, Puro, TK and DT boxes indicate location of PGK-Neo^r^, PGK-Puro^r ^, HSV-TK and diphtheria toxin selection markers. Positions and direction of the genotyping primers PA, PB, PC and PD are indicated. B. Southern blots of targeted ES cell clones with probes from outside the targeting constructs. Lanes 1 and 2, *Nco*I digests were hybridized with probe UL located upstream of the upstream targeting construct. Lane 1, wild-type ES cells (WT); lane 2, a recombinant ES cell clone (B41) targeted with the upstream construct. The WT allele generates a 19.5 kb band, and the recombinant fragment is 9.5 kb. Lanes 3-6, *Stu*1 digested DNA was probed with probe DR located downstream of the downstream targeting construct. Lanes 3 and 5, clone B41; lane 4, C1, a recombinant clone after transfecting B41 with the downstream targeting construct; lane 6, clone D1, derived from C1 after Cre recombinase-mediated recombination. The WT allele, represented by the upstream targeted clone B41, generates a band of >16 kb, and the recombinant fragments are 6.5 kb in C1 (a doubly-targeted 2-loxP clone) and 10 kb in D1 (a 1-loxP clone with the ∼150 kb deletion), respectively. C. Genotyping of 1-loxP ES cells by multiplex PCR with primers PA, PC and PD. The WT band is 435 bp, the amplification product of primers PC and PD; and the deleted allele is 327 bp, a product of amplification between PA and PD. Lanes 1 and 2 represent two clones after Cre mediated deletion (D1 and D5); Lane 3, wild-type ES cells; lane 4, doubly targeted 2-loxP ES cell clone (C1). H_2_O: no template negative control; MW: molecular weight marker. D. Quantitative real-time RT-PCR study for expression of gene elements in PWS critical region. DNaseI treated total brain RNA from 4-week old mice (n = 9 WT, 7 Del) was used as template for qRT-PCR assays. Each sample was assayed in triplicate. *Gapdh* expression was used as internal control. The levels for each sample are normalized to the average of the WT samples. Error bars, standard error of the mean (SEM). Asterisks indicate significant differences; for *Snord64*, *P*<0.0005; *Snord116*, *P*<1 ×10^−7^, and *Snord115*, *P*<0.01.

In addition to *SNORD116,* there are several other C/D box snoRNAs in the interval between *SNRPN (*small nuclear ribonucleoprotein N, GeneID: 6638) and *UBE3A* (ubiquitin-protein ligase E3A, GeneID**:** 7337) in the PWS deletion region [4], [7], [9]. Another cluster of C/D box snoRNA, SNORD115 (formerly known as *HBII-52,* GeneID: 692218) is located downstream of *SNORD116* in both human and mice (Fig. 1A). *SNORD115* has a target binding sequence complementary to serotonin receptor 2C pre-mRNA, and has been reported to influence the regulation of alternative splicing [11]. The lack of PWS phenotypes in individuals or mice lacking *SNORD115* expression, however, excluded these snoRNAs as candidate genes for PWS [4], [12], [13]. Three single-copy snoRNAs, *SNORD107* (*HBII-436,*GeneID: 91380), *SNORD64* (*HBII-13* GeneID: 347686), and *SNORD108* (*HBII-437,* GeneID: 338427) are located upstream of *SNORD116* and were also excluded from playing a role in PWS [5]. In the human genome, there are two copies of *SNORD109* (*HBII-438),* one located upstream of *SNORD116* (*SNORD109A,* GeneID: 338428) and the other downstream of *SNORD115 (SNORD109B*, GeneID: 338429). We were unable to identify a mouse homolog of *SNORD109*. Therefore, lack of expression of the S*NORD116* cluster is most likely responsible for many aspects of PWS.

The PWS deletion region is conserved on mouse chromosome 7C. Prior mouse models for PWS include three different strains of deletion mice that affect expression of multiple genes including the snoRNA region. Imprinting-center (IC) deletion mice [14] and mice with a transgene-induced 6.8 Mb genomic deletion that includes the mouse equivalent of the entire PWS region [15], [16] lack expression of multiple genes and die soon after birth. Occasional survivors with the IC deletion on a hybrid strain background remain small, are fertile and do not become obese [17]. While the knock-out of *Snrpn* alone has no abnormal phenotype [14], a partial deletion of the PWS orthologous region, extending from *Snrpn* to *Ube3a,* removes the expression of the *Snrpn* coding transcript and of all the paternally expressed non-coding elements in between *Snrpn* and *Ube3a* [18]. These mice suffer severe postnatal growth retardation, and 80% die before weaning. Survivors grow to adulthood without obvious defects, despite their smaller size, and do not develop obesity. No metabolic or behavioral studies of these mice have been reported. The high lethality rate in young pups makes phenotypic characterization of adult mice difficult.

In this study, we test the hypothesis that the *SNORD116* snoRNA cluster is the major contributor to the PWS phenotype. To elucidate the role of this snoRNA, we created a new mouse model for PWS by deleting all the copies of *Snord116*. Mice with paternally inherited *Snord116* deletions have striking growth delay in the first three postnatal weeks, but no lethality, which enabled us to carry out multiple neurobehavioral and metabolic tests. *Snord116* deletion mice have normal muscle strength but a defect in motor learning. Adult mice have hyperphagia, elevated levels of plasma ghrelin and altered metabolism, but normal energy homeostasis regulation. Based on our results, we propose that the key defect underlying the hyperphagic behavior in PWS is not defective regulation of energy homeostasis as had been proposed [19], [20], but rather defective control of meal termination. This is the first *in vivo* study of the biological function of a snoRNA in a vertebrate system, and we report the function of this imprinted C/D box snoRNA on early postnatal growth, motor learning and feeding regulation.

## Results

### Creation of the *Snord116del* mouse model

To generate mice deleted for the imprinted *Snord116* snoRNA cluster, we used two chromosome-engineering strategies in parallel, with Cre-mediated deletion either *in vivo* in the ovary, or in cultured male embryonic stem (ES) cells. First, we cloned the genomic sequences from mouse bacterial artificial chromosomes (BAC) RP23-371M8 and RP23-309E15 and made two targeting constructs flanking the *Snord116* cluster (Fig. 1A). By homologous recombination in BRUCE4 ES cells (derived from male blastocysts of the C57BL/6 strain), we inserted two cassettes with loxP sites and drug resistance markers flanked by different Frt sites, upstream and downstream of the *Snord116* cluster (Fig. 1A,B). To obtain 2-loxP ES cells, we deleted the drug resistance markers by transfecting the doubly resistant ES cells with a vector expressing Flp recombinase. To derive 2-loxP mice, 2-loxP ES cells were injected into C57Bl/6J –Tyr^c-2J/J^ albino blastocysts and three male chimeric mice were obtained, with 20–80% ES cell origin based on coat color, all of which transmitted the modified allele through the germ-line. The 2-loxP mice with a paternally derived modified allele were indistinguishable from their wild-type (WT) littermates.

Heterozygous 2-loxP mice were mated to a transgenic strain expressing Cre recombinase under the control of the ovary-specific Zp3 promoter on a C57BL/6J background. When we mated doubly heterozygous (2-loxP/+; Zp3-Cre/+) females with WT C57BL/6J males, we obtained six 1-loxP mice among 20 offspring. The presence of the large deletion was documented by Southern blot (data not shown) and confirmed by PCR amplification of a small product, of expected size and sequence, with primers spanning the ∼150 kb genomic region that is deleted (Fig. 1C).

In the second approach, we generated 1-loxP ES cells with the ∼150 kb deletion by transfecting doubly-targeted ES cells with a Cre recombinase expressing vector. We obtained two male chimeric mice, with 10% and 40% 1-loxP ES-cell derived coat color, respectively, but only the higher-level chimera passed the deletion allele through the germ-line. The 1-loxP mice derived either from 1-loxP ES cells or from the mating of the 2-loxP/Zp3-cre mice exhibited the same degree of growth retardation when the deletion allele was paternally inherited.

### Expression of genes in PWS critical region (PWCR)

The ∼150 kb *Snord116* deletion could potentially change the chromatin conformation of the flanking region or remove regulatory elements and, thus, affect the expression of neighboring genes. To examine this possibility, we measured the expression of gene elements in the region by quantitative RT-PCR of brain samples (Fig. 1D). Compared to WT levels, expression of the upstream snoRNA *Snord107* was decreased by 35±6% (*P*<0.0005), and that of the downstream *Snord115 (MBII-52)* snoRNA cluster was increased by 33±8% (*P*<0.01). Expression of gene elements located further upstream, *Ndn (*Necdin, GeneID: 17984*)* and *Snrpn*, or downstream (*Ube3a*) was unchanged. Although *Ube3a* is expressed exclusively from the maternal allele in brain and, therefore, should not be affected by the *Snord116* deletion on the paternal chromosome, a reduction of the paternal-specific antisense transcript Ube3aAS could potentially affect silencing of the paternal *Ube3a* allele and, thus, change its transcript level. As expected, the expression of *Snord116* was completely abolished, confirming that we have successfully removed all copies of this snoRNA.

### Postnatal growth deficiency and reduced IGF-I

**Figure 2:**
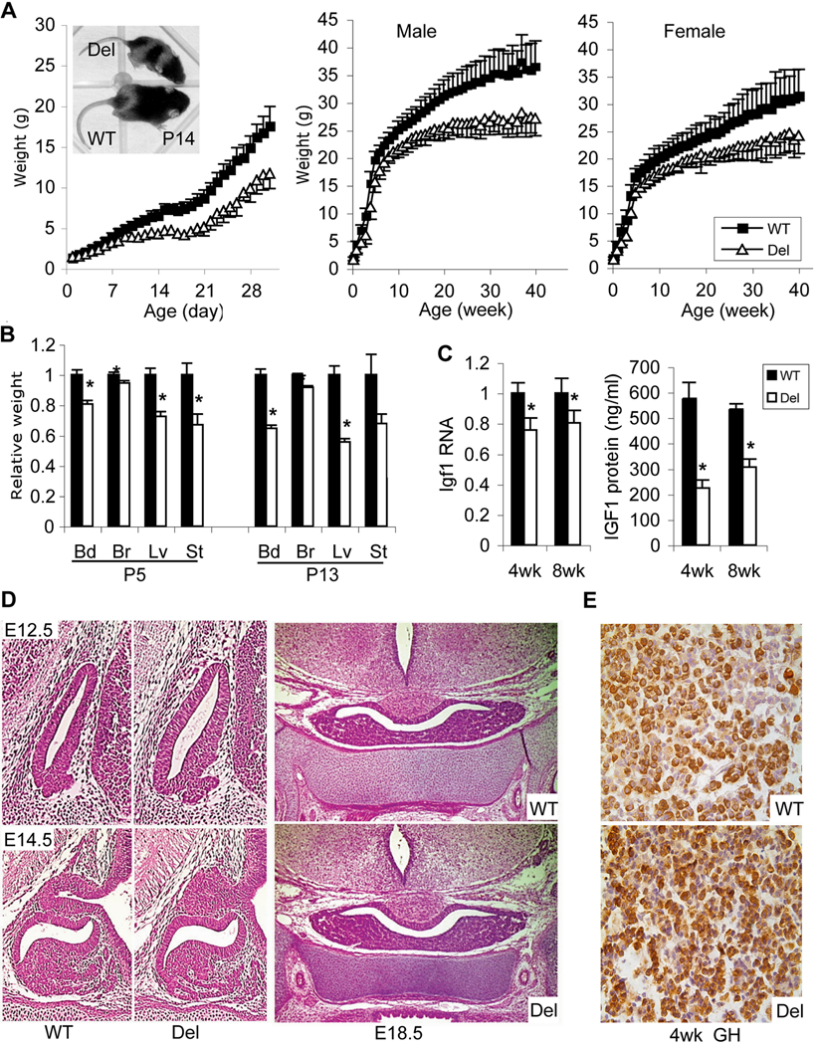
Growth and somatic development of *Snord116del* mice. A. Daily weight curves for males (left) and weekly weight curves for males (middle) and females (right). The female weight curve in the first month (not shown) overlaps with the male curve. Error bars, standard deviations. Significant weight differences between *Snord116del* mice and their WT littermates ranged from *P*<0.05 in young pups, after postnatal day 2 (P2), to *P*<1 ×10^−8^ after 6 months (n = 15∼20 in each sex/genotype group). Insert photo: *Snord116del* (top) and WT (bottom) pups at P14. B. Comparison of body and organ weights in *Snord116del* mice and WT littermates at P5 and P13 (P5: n = 27 WT, 24 Del; P13: n = 8 WT, 9 Del). Error bar: SEM. At P5, all differences were significant at *P*<0.003 except for brain at *P*<0.02. At P13, all differences were significant at *P*<0.0001 except for stomach, *P* = 0.05. Bd: body, Br: brain, Lv: liver, St: stomach. C. Levels of *Igf1* transcripts in liver and IGF-I protein in serum are reduced in deletion mice. Left: Liver RNA from 4-week old (n = 9 WT, 7 Del) and 8-week old (n = 8 WT, 8 Del) mice were assayed by qRT-PCR with *Gapdh* levels as internal control. WT levels were set at 1. Error bar: SEM. Asterisks: *P*<0.05. Right: Serum levels of IGF-I protein from the same group of mice were measured by ELISA. Asterisks: *P*<0.0005. D. Hematoxylin and eosin stained sagittal sections of the pituitary at E12.5 and E14.5 (left WT, middle Del) and coronal sections at E18.5 (right) revealed normal pituitary size and structure. E. Immunohistochemical staining for growth hormone (somatotrophs) revealed no detectable differences in WT (top) and Del (bottom) mice at 4 weeks of age.

We observed severe postnatal growth retardation in mice with paternally inherited *Snord116* deletion (Fig. 2). Mice with maternal inheritance of the *Snord116* deletion grew normally (data not shown), consistent with the fact that the *Snord116* cluster is imprinted and the maternal copies are silenced. From now on, “deletion mice”, “*Snord116del* mice” or “mutant mice” refer to mice with a paternal deletion.

At prenatal stages E12.5, E14.5 and E18.5, the mutant embryos and placenta were of normal size (data not shown). At birth, deletion mice were also indistinguishable from WT. As of postnatal day 2 (P2), however, they failed to gain weight as effectively as their WT littermates, and by 3 weeks of age, the mutants' weight was ∼60% of the WT weight for males (Fig. 2A, left) and females (not shown). Although the mutants' rate of growth appeared to normalize after weaning, the weight differences persisted to adulthood for both genders (Fig. 2A, middle and right). The length of the mutant mice was also decreased. At 5 months of age, the length of the mutant males was 95.8±0.4% of WT (n = 13 WT, 9 Del, *P*<1×10^−6^), and the length of the mutant females was 97.6±0.8% of WT, (n = 12 WT, 12 Del, *P*<0.05), as measured under anesthesia from the tip of the nose to the anus. Unlike previously reported PWS mouse models with neonatal lethality, almost all the *Snord116del* mice survived to adulthood, and appear healthy at their current age of 18 months.

Possible causes of the striking early postnatal growth failure include reduced milk intake. Human neonates with PWS have severe hypotonia and are unable to suck effectively, so that gavage feeding is usually required. In a previous mouse model for PWS, decreased milk intake and impaired righting ability from the prone position had been reported [18]. In contrast, we observed sufficient milk in the stomachs of young pups with *Snord116* deletion when their abdomens were still transparent. Also, the mutant pups were not hypotonic. When tested at P5, they accomplished the righting task with equal or better efficiency than their WT littermates, and they could hold on to a 45 degree rough surface longer than the WT littermates (data not shown). A careful dissection at P5 and P13 showed strikingly reduced sizes of liver (72±15% of WT at P5 and 56±2% at P13; both *P*<0.0001), and stomach (67±36% at P5 and 68±6% at P13; *P*<0.05 and *P* = 0.05, respectively), but brain weights were only slightly decreased (95±7% of WT at P5, and 92±1% at P13; both *P*<0.02) (Fig. 2B). The disproportionately small stomach size at P5 may suggest that reduced food intake could contribute to the growth retardation. We also observed decreased subcutaneous fat storage during the dissection. Histologic examination of H & E stained sections of all other inner organs, including the brain, revealed no apparent abnormalities in the *Snord116del* mice compared to WT littermates.

Since individuals with PWS have short stature after early childhood that is in part due to growth hormone (GH) deficiency, and the growth retardation of *Snord116del* mice persisted to adulthood, we measured the direct downstream effector of GH action, the transcript levels of insulin-like growth factor (*Igf1,* GeneID: 16000) in liver. By performing quantitative RT-PCR with liver RNA samples from 4-wk and 8-wk old mice, we found that *Igf1* mRNA expression was significantly lower in the mutants at both time points, 76±8% of the WT level at 4 wk and 80±8% at 8 wk (both *P*<0.04) (Fig. 2C left). Measurements of circulating IGF-I protein in serum showed an even more drastic reduction to 39±5% of the WT level at 4 wk and 57±6% at 8 wk (both *P*<0.0005) (Fig.2C right). These results indicate involvement of the GH pathway in the reduced growth of the mutants.

### Normal structure and development of pituitary

Since we observed a reduction in liver *Igf1* mRNA expression and in circulating levels of IGF1 protein, indicative of GH deficiency, and since most individuals with PWS are GH deficient, we studied the structure and development of the pituitary gland to determine whether the GH deficiency is caused by a structural pituitary defect or a reduction in the number of GH producing cells in the anterior pituitary. We performed H&E staining of E12.5, E14.5 and E18.5 embryonic and adult (4 week old) pituitaries. The developing pituitary gland appeared morphologically normal in sections from *Snord116del* embryos (Fig. 2D). To obtain quantitative data, we determined the volume of the pituitary gland at E18.5 and at 4 wk by using three-dimensional reconstruction. The volume of the pituitary gland was not significantly reduced in E18.5 *Snord116* embryos compared to their WT littermates. For example, at E18.5 pituitary volume was 5.1×±0.8×10^6^ A.U.^3^, (n = 3 WT) compared to 5.0±0.9×10^6^ A.U.^3^ (n = 3 Del). In adult *Snord116del* mice (n = 3), the volume of the pituitary gland was also not significantly reduced, relative to body weight (data not shown).

Given that the morphology of the *Snord116* pituitary was normal, we asked whether the GH deficiency observed could be due to a reduction in hormone-producing cells in the anterior pituitary. To address this question, we performed immunohistochemical staining for the five hormone-producing cell types present in the anterior pituitary. This analysis revealed that all cell types are present in normal proportions in the developing (E18.5) and adult (P28) *Snord116* pituitary compared to the WT (Supplementary Figure S1). With no reduction in the number of GH-producing cells (somatotrophs, Fig. 2E) and apparently normal immunogenicity of the GH protein in these cells, the cause of GH-deficiency most likely resides in the regulation of GH release.

### Normal fertility and delayed sexual maturation

Individuals with PWS have hypoplastic genitalia, and delayed and often incomplete sexual maturation, secondary to deficiency of gonadotropic hormones. In *Snord116del* mice, the sizes of testes and ovaries were proportional to their body size and histologically normal. The fertility of male and female mutant adults was normal. The males consistently plugged their female partners, and the females gave birth to litters of 4–8 pups almost every month until 12 months of age, when the mating was stopped. This breeding profile is typical of C57BL/6 mice [21]. As an indicator of sexual maturation, we studied the timing of vaginal opening in *Snord116del* females and WT littermates. In the mutants, vaginal opening was delayed by 3.6±0.7 days (n = 39 WT, 27 del, *P*<0.001). These results indicate delayed sexual maturation, consistent with the human PWS phenotype.

### Increased anxiety and normal working/spatial memory

People with PWS have developmental delay, motor and cognitive deficits, as well as anxiety and behavioral abnormalities. We, therefore, subjected *Snord116del* mice and their WT littermates to a battery of neurobehavioral test at 2–6 months of age.

**Figure 3:**
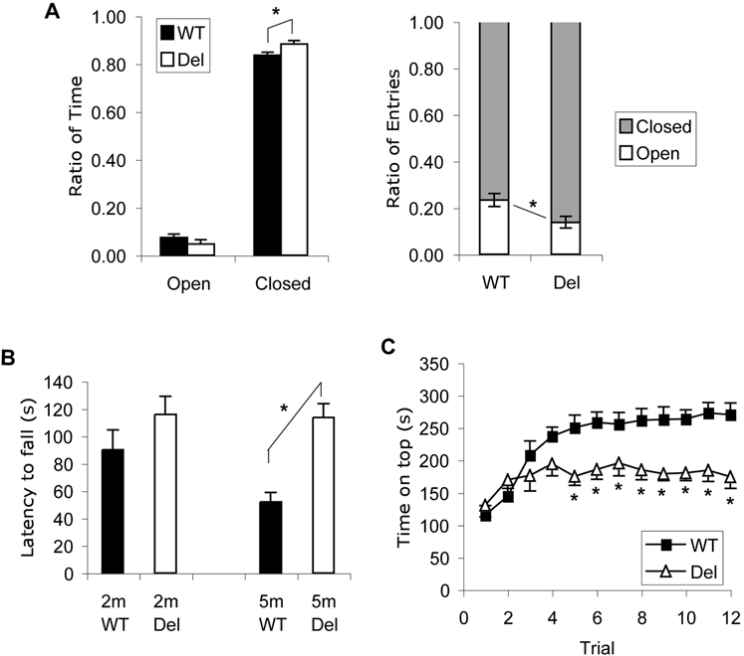
Neurobehavioral tests of *Snord116del* and WT littermates. A. Elevated plus maze. Mice were tested at 3 months of age (n = 15, both genotypes). Left panel: *Snord116del* mice spent more time in the closed arms (*P*<0.05). Right panel: mutant mice made more entries to the closed arms (*P*<0.02). B. Wire-hanging test. At 5 months of age, *Snord116del* mice were able to hang significantly longer than the WT littermates (*P*<5 ×10^−5^, n = 17, both genotypes). At 2 months of age, the difference was not significant (n = 13 WT, 9 Del). C. Rotarod test of balance and motor learning. Mice were tested at 2 months (n = 18 WT, 10 Del). Two trials were done on each day for 6 consecutive days. Asterisks indicate significant differences between WT and deletion mice (*P*<0.05). All error bars are SEM.

In the Open Field test, a measure of exploratory behavior and general anxiety, we detected no difference between the deletion mice and WT littermates in total distance traveled, total time of rearing and grooming, time spent in the inner square, and frequencies of urination and defecation (data not shown). When tested in the Elevated Plus Maze at 3–4 months of age, the deletion mice made more entries into the closed arms (77±3% WT, 86±2% Del, *P*<0.02), and spent more time in the closed arms (84±2 WT, 88±1 Del, *P*<0.05) (Fig. 3A). We repeated this test with a different cohort of mice at a similar age (n = 21 WT, 19 Del), and obtained the same results, with the deletion mice having a significantly higher ratio of entries into the closed arms (77±2% WT, 89±2% Del, *P*<0.0001) and a trend of longer times spent in the closed arms (76±3% WT, 83±3% Del, *P* = 0.08). These results indicate that the *Snord116del* mice have an elevated level of anxiety or fear.

To assess learning and memory of the *Snord116del* mice, we measured their performance in the Y maze at 3 months of age. The rates of spontaneous alternation were identical in the mutants and their WT littermates, Del 59±3% (n = 17) and WT 58±2% (n = 17), indicating no difference in short-term working memory. For the novel arm preference test, we used a different cohort of mice at 5 months of age. The *Snord116del* mice performed as well as their WT littermates, with 40±1% of entries into the novel arm for the WT mice (n = 9), and 41±1% for the mutants (n = 11). These two types of results indicate that the *Snord116del* mice do not have any obvious defect in working memory or spatial memory.

### Normal motor function and defect in motor learning

To evaluate their muscle tone and strength, *Snord116del* mice and their WT littermates were subjected to a wire-hanging test. To our surprise, at 5 months of age, the mutants outperformed the WT mice (Fig. 3B). While the latency to fall was 52±7 sec for the WT, the mutants were able to hold on for 114±10 sec (*P*<4×10^−5^). At this age, however, the WT mice weighed considerably more (30.7±0.6 g) than the mutants (25.0±0.4 g). We, therefore, tested another cohort at 2 months of age. The latencies were not significantly different: 90±15 sec for WT and 116±13 sec for mutant littermates (Fig. 3B); and the body weights at 2 months were also more similar, with 24.4±0.7 g for WT and 21.0±0.5 g for mutants. We conclude that the *Snord116del* mice are definitely not hypotonic, and that their reduced body weight may have contributed to their better performance in the wire-hanging test at 5 months of age.

To assess motor coordination and balance, as well as motor learning, we measured the performance on an accelerating rotarod at 2 and 5 months of age. We applied two trials per day for 6 days. In the first trial on day 1, the 2 months old *Snord116del* mice showed a similar level of performance as their WT littermates (Fig. 3C), and at 5 months of age, they outperformed the WT mice (*P*<0.004, data not shown). This is consistent with their performance in the wire-hanging test at these ages. During the six days of training, the WT mice at both ages improved significantly. In contrast, the learning curves of the mutants remained essentially flat, indicating that they have a motor learning deficiency (Fig. 3C and data not shown).

### Normal sensitivity to pain

Individuals with PWS have a higher pain threshold and decreased pain sensitivity compared to unaffected controls [2]. To evaluate the nociception and response time to a painful stimulus in 6-month old mice, we used a hot plate test. When placed on a 52.5°C metal surface, mice of both genotypes had identical response times. The WT mice (n = 17) had a response latency of 8.7±0.6 sec, and their *Snord116del* littermates (n = 17) had a latency of 8.8±0.6 sec. Thus, the *Snord116* deletion did not cause decreased sensitivity to thermal pain.

### Feeding behavior and body composition on high-fat diet

Characteristically, individuals with PWS develop hyperphagia at 2–6 years of age due to an obsession with food, which leads to profound obesity and type 2 diabetes when their access to food is not restricted. In contrast to the human PWS phenotype, the *Snord116del* mice maintain a stable body weight as adults, indicating proper feeding regulation under normal conditions. We, therefore, studied their feeding behavior under different challenges.

**Figure 4:**
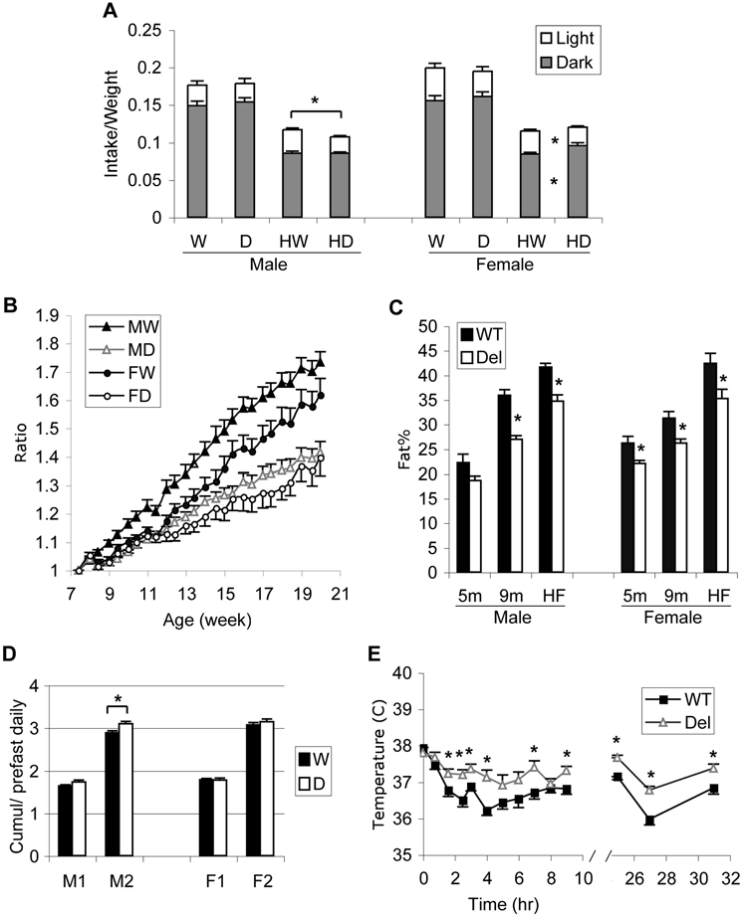
Feeding and energy homeostasis regulation of *Snord116del* mice and their WT littermates. A. Daily food intake and response to High-Fat (HF) diet. The intake of rodent chow during the dark (black bars) and light cycles (open bars) were measured for five consecutive days at 7 weeks of age. Then, the mice were placed on HF diet, and their intake was measured daily for five consecutive days. Food intake was normalized to average body weight during the five days of measurement. The daily intake on regular chow diet was not different between WT (W) and *Snord116del* (D) mice. On HF diet, the daily intake of the deletion males (HD) was significantly lower than that of their WT littermates (HW) (*P*<0.005). This was entirely due to decreased daytime intake. The deletion females on HF (HD) had lower intake in the dark (*P*<0.05), which was compensated by increased daytime intake (*P*<0.05), so that their total daily intake was not different from their WT littermates (HW). (Males, n = 16 WT, 16 Del; females n = 15 WT, 11 Del). B. Deletion mice gain less weight on HF diet. Eight-week old mice were put on HF for 4 months, and their body weight was measured twice per week and plotted as the ratio to their weight prior to HF diet. Differences between male WT (MW, n = 16) and male *Snord116del* (MD, n = 16) mice were significant at *P*<0.01 except for the first week on HF. Differences between female WT (FW, n = 15) and female deletion (FD, n = 11) mice were significant at *P*<0.05 after 7 weeks on HF. C. Deletion mice have less body fat on regular and HF diet. Dual energy X-ray absorptiometry was used to measure body composition of 5-months and 9-months old mice on rodent chow, and 6-months old mice that had been on HF diet for 4 months. The differences between WT and *Snord116del* mice are all significant, except for the 5-months old males on chow. Males, at 5 months (n = 13 WT, 9 Del; *P* = 0.07); at 9 months (n = 17, both genotypes, *P*<2 ×10^−7^); at 6 months after 4 months on HF diet (n = 16, *P*<0.0001). Females at 5 months (n = 12, both genotypes, *P*<0.02), at 9 months (n = 14 WT, 10 Del; *P*<0.007) and at 6 months after 4 months on HF diet (n = 15 WT, 10 Del; *P*<0.02). D. Compensatory food intake after fasting. Mice at 3 months of age were fasted for 24 hr, and the ratio of their cumulative food intake to their average pre-fasting daily intake was computed. M1 and M2, cumulative intake ratios of males in the first 24 and 48 hr (n = 13 WT, 9 Del). F1 and F2, cumulative intake ratios of females in the first 24 and 48 hr (n = 12). The deletion mice were able to compensate within 48 hr for the missed feeding. The deletion males over-compensated compared to their WT littermates (*P*<0.02). E. Maintenance of body temperature in cold environment. The core body temperature of 4 months old *Snord116del* (n = 9) and WT (n = 13) males in a 4°C cold room was measured with an anal probe. The mutants were better able to maintain their core temperatures over extended time. Asterisks indicate *P*<0.05.

First, we measured their food intake on regular rodent chow (14% calories from fat, 3.0 kcal/g). After one week of single housing, food intake was measured for five consecutive days. The 7-week old *Snord116del* mice (D) and their WT littermates (W) consumed the same amount of food in both dark and light phases, when intake was normalized to body weight (Fig. 4A).

Next, we provided the mice with a high fat (HF) diet (45% calories from fat, 4.73 kcal/g), and measured their food intake over five consecutive days. Both the WT (HW) and *Snord116del* mice (HD) reduced their total food intake on the HF diet (Fig. 4A). Compared to their caloric energy intake on rodent chow, the WT and deletion males adjusted their daily energy intake on HF food to 104.4±2.4% and 94.6±1.6%, respectively, with the deletion males consuming less than their WT littermates (−8±1.5%, *P*<0.005). This difference is due to less daytime intake (*P*<0.006). The females' energy intake was not significantly different, 91.0±2.1% for WT and 97.1±2.5% for mutants. But like the deletion males, the deletion females had lower intake in the light phase (*P*<0.04) that was compensated by higher intake in the dark phase of the daily cycle (*P*<0.03).

To determine whether long-term HF diet would result in obesity, we kept the *Snord116del* mice and their WT littermates on the HF diet from 8 weeks of age, and plotted their weight changes during the next 4 months relative to the average weight at 7 weeks of age (Fig. 4B). Both male and female deletion mice gained less weight than the WT controls. Thus, they were relatively more resistant to HF diet-induced obesity. The effect was more pronounced in the mutant males, consistent with their lower energy intake on HF diet as shown in Fig. 4A.

PWS is associated with high body fat mass and low muscle mass. We measured total fat and lean body mass ratio in the *Snord116del* mice by Dual Energy X-ray absorptiometry. After 4 months on the HF diet, the *Snord116del* mice had significantly lower body fat content than WT littermates (−6.9%, *P*<0.0001 for males and −7.2%, *P*<0.02 for females) (Fig. 4C, HF). A cohort of mice kept on regular rodent chow diet for 5 months, showed an insignificant trend of lower fat content in deletion males compared to their WT littermates (−3.6%, *P* = 0.07), and a significantly lower fat content in deletion females (−4.2%, *P*<0.02). A group of 9 months old mice on regular rodent chow showed the same difference, with significantly lower body fat content in deletion males (−9%, *P*<2×10^−7^), and females (−5%, *P*<0.02) (Fig. 4C). Taken together, these results indicate that the *Snord116* mice are leaner that WT on a regular diet, as well as on a HF diet. The fact that the *Snord116del* mice on HF diet have a higher body fat content than those on the regular diet confirms that they are capable of anabolic fat storage. Under both dietary regimes, however, the overall fat storage is much lower in the mutant than in the WT mice, which is contrary to the human PWS phenotype.

Finally, we assessed the ability to adjust food intake after fasting. After a 24 hr fast, normal mice regain their pre-fasting body weight within 48 hr due to compensatory hyperphagia. Some obese strains, such as *ob/ob* (GeneID: 16846) mice or those with neuronal ablation in the hypothalamic feeding circuit [22], have abnormal weight regulation and are unable to compensate for missed meals and to regain their body weight within 48 hr. Therefore, we tested 3 months old *Snord116del* mice and their WT littermates for their ability to compensate. After a 24 hr fast and re-feeding, both the deletion and WT mice showed similar food intake, as measured at 2, 4, 8, 12, 24, 48, and 72 hr after the fasting period, and mice of both genotypes gained back the weight they lost during fasting within 48 hrs (data not shown). When we measured the 48 hr cumulative food intake, deletion males (M2) showed a significantly higher compensatory intake, 3.09±0.06 units of the pre-fasting daily intake, than the WT males, with only 2.88±0.05 units of daily intake (*P*<0.02) (Fig. 4D). This difference may reflect the known insufficient compensatory feeding of the WT C57BL/6 males, which are prone to diet-induce obesity[23], because it was not seen in females. The 48 hr cumulative food intake for females (F2) was not significantly different, with WT and deletion mice taking in 3.07±0.05 units and 3.14±0.06 units of their pre-fasting daily intake, respectively. We conclude that normal feeding regulation allows the *Snord116del* mice to restore missed meals and maintain body weight with good precision.

### Normal thermal regulation

In addition to abnormal re-feeding response to fasting, *ob/ob* mice are also defective in thermal regulation. Within one hour of exposure to a cold environment at 4°C, their core body temperature drops by more than 3.5°C, and within 2–4 hours, it reaches a dangerous range of hypothermia at ∼27°C [24], [25]. We tested 5 month old WT and *Snord116del* mice for their ability to maintain body temperature when placed in a 4°C room. The deletion mice were able to keep their core body temperature for the entire testing period of 32 hours (Fig. 4E), with the core temperature of the deletion males roughly 0.5°C higher than that of their WT littermates for most of the tested time points (*P*<0.05). The deletion females were also able to keep their core body temperature in the cold with slightly higher levels than the WT (less than 0.5°C) at some testing points (data not shown). Given their smaller size and lower body fat mass, the deletion mice may require a slightly higher core temperature to maintain whole body temperature. In summary, we conclude that the *Snord116del* mice are capable of precise thermal regulation in the cold.

### Insulin sensitivity is normal in females and increased in males

**Figure 5:**
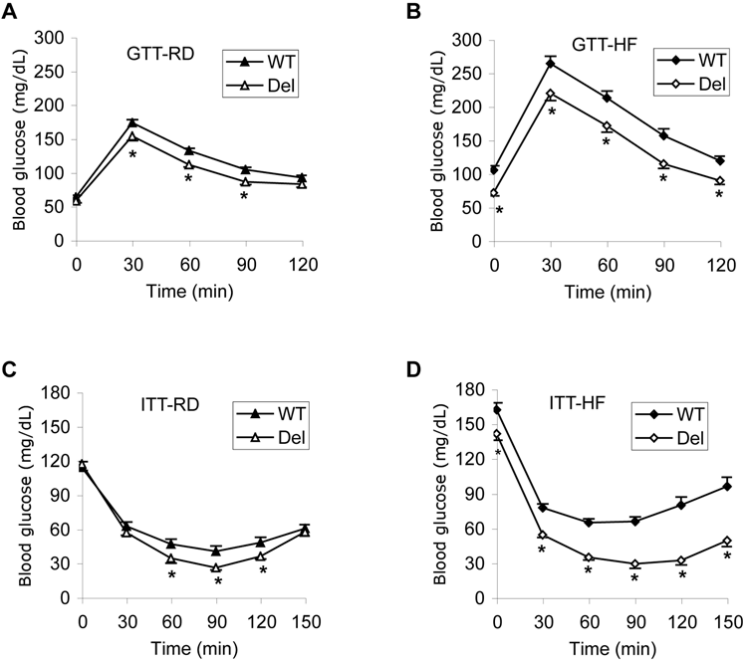
Glucose and insulin tolerance tests of male mice. A. Glucose tolerance test (GTT) for mice on regular diet (RD) at 6 months of age. Mice were injected with 1 g/kg glucose at time zero after 16 hr overnight fasting and serum glucose was measured for up to 2 h. n = 13 WT, 9 Del). Deletion mice were better able to clear the glucose that WT, *P*<0.05 at 30 min, and *P*<0.001 at 60 and 90 min time points). B. Glucose tolerance test for mice after 4 months on HF diet (n = 16) starting from 8 weeks of age (*P*<0.01 for all time points). C. Insulin tolerance test (ITT) for mice on regular diet (RD) at 6 months of age. Wild type (n = 13) and mutant (n = 9) mice were injected with 1.6 U/kg insulin at time zero after 5 hr fasting from the onset of the light cycle (*P*<0.05 at 60, 90 and 120 min). D. Insulin tolerance test for 6-months old mice after 4 months of HF diet. WT mice (n = 15) and deletion mice (n = 16) were injected with 2 U/kg insulin (*P*<0.02 at time 0, and *P*<0.0002 at all other time points). Deletion males were more sensitive to insulin than WT.

Individuals with PWS and obesity may have insulin insensitivity, but it is less pronounced than in subjects with simple obesity [26]. We performed glucose and insulin tolerance tests on 6 months old mice after 4 months on HF diet, and on a similarly aged group on regular diet. The female *Snord116del* mice and their WT littermates showed no significant differences in their responses, either on the HF or on the regular diet (data not shown). The *Snord116del* males on the regular diet had the same level of basal blood glucose as WT, but a lower peak after glucose injection (Fig. 5A). They also responded more dramatically to insulin injection, indicating increased insulin sensitivity (Fig. 5C). The deletion males on the HF diet had lower resting glucose levels and a smaller peak after glucose injection (Fig. 5B), as well as a greatly increased sensitivity to insulin (Fig. 5D). Taken together, the resistance to obesity of the *Snord116del* males correlates with increased sensitivity to insulin.

### Metabolic chamber studies reveal hyperphagia

**Figure 6:**
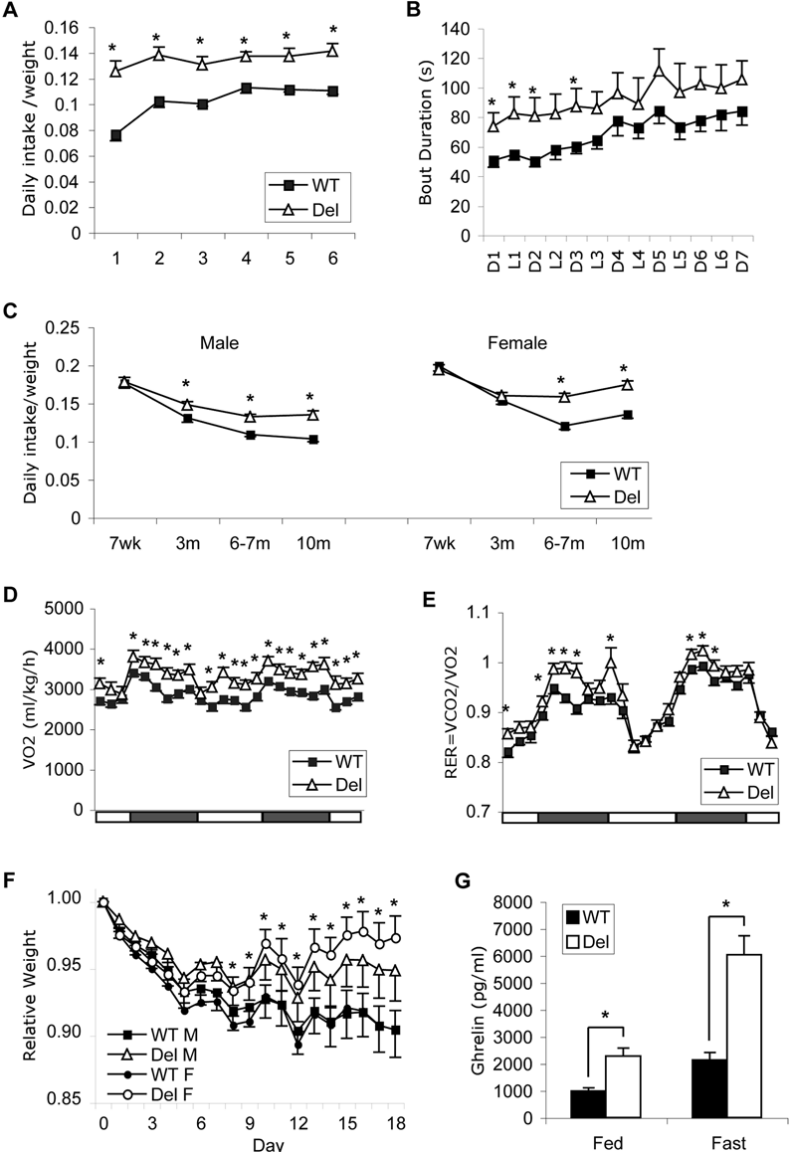
* Snord116del* mice are hyperphagic and have altered metabolism. A. Daily food intake during 6 days in Oxymax metabolic chambers was normalized to body weight (n = 8 WT, 7 Del, all points *P*<0.05). Error Bars: SEM. B. Average feeding bout duration of the same group of mice as in (A) during each 12 hr cycle. D, dark cycles; L, light cycles. Asterisks indicate *P*<0.05. C. Daily food intake at different ages. Daily food intake was averaged over 5–7 consecutive days and normalized to body weight of each individual. At 7 week, males n = 16 both genotypes; females, n = 15 WT, 11 Del; at 3 months, males n = 13 WT, 9 Del, *P*<0.04; females n = 12 WT, 12 Del; at 6–7 months, (males n = 15 both genotypes, *P*<2 ×10^−6^; females n = 14 WT, 16 Del, *P*<3 ×10^−6^, and at 10 months males n = 21 WT, 19 Del, *P*<3 ×10^−5^; females n = 13 both genotypes, *P*<2 ×10^−5^. D. Oxygen consumption rates (averaged over 2 hr time periods) on the second and third day in the metabolic chamber (n = 15 both genotypes). Bars at bottom indicate light and dark cycles. Asterisks, *P*<0.05. E. Respiratory exchange rate (RER) averaged over 2 hr time periods for the same group of mice as in (D) during the same time in the metabolic chamber. Asterisks, *P*<0.05. F. Weight change in mice food-restricted to 80% their normal daily intake. Males (n = 11 both genotypes) and females (n = 13 both genotypes) at 11 months of age. Asterisks indicate significant difference between WT and Del females with *P*<0.05. G. *Snord116del* mice have increased ghrelin levels. Plasma ghrelin levels were measured in *ad lib* fed mice at 11 months of age (n = 12 both genotypes; *P*<0.002) and 24 hr fasted mice (n = 13 both genotypes ; *P*<0.0002)

To determine why the *Snord116del* mice are resistant to developing obesity, we carried out more detailed analyses of their feeding behavior and metabolism. We placed 6 months old *Snord116del* males and their WT littermates into metabolic chambers for 7 days. To our surprise, the mutant mice consumed more food than their WT littermates after normalizing intake to body weight (Fig. 6A). The water intake mirrored the food intake with the deletion mice consuming more water as well (data not shown). This result is apparently inconsistent with our previous finding that 7 week old *Snord116del* mice had the same daily food intake, after normalizing to body weight, as their WT littermates when measured in their home cages (Fig. 4A). Suspecting that the stress of the metabolic chambers could be the cause of the observed difference, we placed the 6 months old mice that had been tested in the chambers back into their home cages and measured their food intake. We found that their daily food intake was the same as during the last three days in the metabolic chambers. The *Snord116del* males (n = 21 WT, 19 Del) showed a 22±3% increase in daily food intake normalized to weight (*P*<2×10^−6^). Deletion females at 6 months of age also had an increased daily intake as measured in metabolic chambers, with a seven day average of a 32±4% increase after normalizing to body weight (n = 14 WT, 16 Del, *P*<3×10^−6^). Taken together, these data confirm that both male and female *Snord116del* mice are hyperphagic at 6 month of age. When the details of their feeding behavior were analyzed, the WT and deletion mice had a similar number of feeding bouts during each light and dark cycle, but the deletion mice had longer bouts, and, therefore, an extended total feeding time (Fig. 6B).

We speculated that the hyperphagia detected in 6-month old mutants could represent a failure to reduce food intake after the rapid growth period, as the 7-week old mice were still growing and had a higher ratio of food intake to body weight than older mice. To evaluate a possible age effect, we measured food intake in 3-month old mice in their home cages. *Snord116del* mice were hyperphagic, but to a lesser degree than at 6 months, with an increased daily food intake of 13±3% (*P*<0.05) in males, and an insignificant increase in females (4.3±2.6%) (Fig. 6C). When tested at 10 months, both male and female *Snord116del* mice were significantly hyperphagic (males 31±5%, *P*<3×10^−5^; females 29±4%, *P*<2×10^−5^) (Fig. 6C). The results document the development of hyperphagia after the initial period of growth which resembles the behavior in PWS.

### Elevated oxygen consumption and respiratory exchange rates

While the *Snord116* deletion mice ate more, they remained smaller and leaner, raising questions about differences in energy expenditure. The locomotor activities of the *Snord116del* mice and their WT littermates were similar in the 7 day metabolic chamber study, with mice of both genotypes showing higher initial activities indicative of a normal response to a novel environment (Supplementary figure S2). Their oxygen consumption rates, however, showed a clear difference, with the deletion mice having higher rates of oxygen consumption (Fig. 6D), part of which could be attributed to altered fuel usage in *Snord116del* mice, as they had a higher Respiratory Exchange Rate (RER) indicating more carbohydrate and less fat usage (Fig. 6E).

The energy expenditure difference, however, cannot fully account for the ∼30% increase in food intake. Therefore, we tested whether *Snord116del* mice truly need the ingested food by restricting their intake to 80% of their normal intake levels. While WT mice gradually lost weight, the *Snord116del* mice were better at maintaining their weight, with females showing significant and males showing a trend of improved weight stability (Fig. 6F). We concluded that the hyperphagia in *Snord116del* mice is not simply due to increased energy requirement.

### Increased circulating ghrelin level

Human PWS individuals have increased circulating levels of ghrelin, a potent orexigenic peptide, which is thought to contribute to their hyperphagic behavior. We, therefore, asked whether the deletion of the *Snord116* cluster in mice causes abnormal secretion of ghrelin as well. As shown in Fig. 6G, ghrelin levels in adult *Snord116del* mice with *ad libitum* access to food are increased to 2.3±0.3 fold compared to WT level (*P*<0.002). This increased level is similar to that seen in normal mice after 24 hr fasting. The deletion mice showed a normal response to fasting, i.e. a proportional increase to 2.8±0.3 fold of the fasted WT level (*P*<0.0002). Our finding of increased circulating ghrelin in the *Snord116del* mice provides a molecular handle towards further dissection of the hyperphagia phenotype.

## Discussion

In our attempt to dissect the PWS deletion region and identify individual genes that contribute to the phenotype, we focused on the previously implicated snoRNA cluster *Snord116* (*Pwcr1/MBII-85*) and created a mouse model that is deleted for this snoRNA cluster. The deletion mice recapitulate a subset of the PWS phenotype, such as postnatal growth retardation, delayed sexual maturation, increased anxiety, motor learning deficit, hyperphagia and hyperghrelinemia (Table 1). PWS features that are lacking include hypotonia, obesity and decreased sensitivity to pain. The differences between humans and mice could be species-specific, or other genes could be involved in combination with the *Snord116* cluster. In this first deletion mouse model for any snoRNA, we identified specific defects in early postnatal growth, feeding behavior and metabolic fuel usage that might provide insight into the pathophysiology of PWS and mechanisms of obesity in general.

**Table 1:** Phenotypes of human Prader-Willi syndrome and *Snord116del* mouse

PWS feature	Mouse feature or test	*Snord116 del* mice	Comparison
**Growth and development**			
Growth delay/short stature	Growth curve	Severe growth retardation	+
GH deficiency	Liver *Igf1* RNA for GH function	↓	+
Hypogonadism	Morphology and histology of gonads	Normal	-
Delayed sexual maturation	Time of vaginal opening in females to assess sexual maturation	Delayed	+
Infertility	Fertility and litter size	Normal	-
**Neurological features**			
Hypotonia	Wire-hanging test and initial rotarod test for motor strength	Normal	-
Learning disability	Rotarod test for motor learning	Deficient	+
Behavior disturbance, anxiety	Elevated plus maze testing for fear and anxiety	↑	+
Proficiency at jigsaw puzzles	Y maze test for working memory and spatial memory	Normal	+
Insensitivity to pain	Hot plate for nociception	Normal	-
**Feeding and energy homeostasis**			
Hyperphagia, with onset at 2–6 yr	Daily food intake	Normal at 7 wk, hyperphagia after 3 mo	+
Longer meal time	Feeding bout duration	↑	+
Obsession with food, lack appetite control	Ability to adjust caloric intake level to high fat (HF) diet	Normal	-
	24hr fasting and re-feeding	Normal	-
Obesity	Long term weight gain on HF diet	Resistant to obesity	O
	Measurement of body fat content	↓	O
Type II diabetes	Glucose and insulin response	F: Normal	+
		M: insulin sensitivity↑	O
Abnormal temperature regulation	Core body temperature in cold	Stable	-
Elevated ghrelin	Radioimmunoassay of plasma ghrelin level	↑	+

+ = concordant; - = discordant; O = phenotype deviates in opposite direction

### Is the lack of *Snord116* causing the observed phenotypes?

The deletion of ∼150 kb of genomic DNA could alter the expression of neighboring or distant loci, for example, by changing the chromatin conformation of the flanking region, or by deleting enhancer/repressor elements or unidentified miRNAs. We have evidence that the observed phenotypes are not due to changes in the expression of nearby genes. First, the expression levels for the closest protein coding genes, *Ndn* (necdin), *Snrpn*, and *Ube3a* in brain were not changed in quantitative real-time RT-PCR assays. Second, a large-scale gene expression profiling study using Illumina microarrays also revealed no changes in transcript levels of nearby protein coding genes and expressed sequence tags (data not shown). Therefore, the *Snord116* deletion does not affect the expression of flanking protein-coding genes in the PWS critical region (PWCR).

The PWCR contains many other snoRNA genes. In the human PWCR, the transcription unit of *SNURF*-*SNRPN* includes all the intron-encoded snoRNAs in a single large RNA precursor [9]. A similar large transcript was recently identified in mouse [27]. Therefore, the *Snord116* deletion could affect the processing efficiency of intronic elements that are located upstream or downstream of the deletion breakpoints. Such a mechanism might explain the small decrease in *Snord107* expression and the small increase in *Snord115* expression that we observed in the brain of *Snord116del* mice. In previous studies, however, *SNORD107* and *SNORD115/Snord115* were excluded from contributing to the PWS phenotype in humans and mice (see Introduction). Therefore, it is unlikely that the subtle changes in the levels of these snoRNAs could be causing the major phenotypes in *Snord116del* mice.

Experimental proof that loss of a specific gene is responsible for a certain phenotype is straightforward for single-copy genes, where a point mutation can be made to abolish gene function without affecting the genomic structure. The fact that *Snord116* exists as a gene cluster of at least 41 copies and spans ∼150 kb of genomic DNA precludes such an experiment. Alternatively, one could test whether a transgene expressing *Snord116* corrects the abnormal phenotype of the deletion mice. We made several transgenic mouse strains expressing *Snord116* from a neural-specific enolase promoter and achieved *Snord116* expression levels comparable to that of an endogenous single-copy gene. Expression from our transgene construct, which contains a single copy of *Snord116* within nucleolin intron 11[28], is comparable to that of an average endogenous single-copy gene, but far lower than the endogenous level of *Snord116* expressed from the >40-copy cluster (data not shown). Consequently, the transgenes failed to rescue the neonatal growth retardation and lethality present in *Snrpn* to *Ube3a* deletion mice [18]. To achieve a higher level of *Snord116* expression, as may be required for transgenic rescue, the creation of BAC transgenics might be an option which, however, would face the risk of introducing unrecognized genetic elements such as miRNAs.

As for the possibility of an unidentified gene or genes in the deleted interval being responsible for the observed phenotypes, we were unable to identify any conserved elements other than the multiple copies of *Snord116* in the deleted interval. The exons of the host gene, which in mouse encode an open reading frame (hypothetical protein LOC664788), are not conserved in human. Our conclusion that the *Snord116* deletion is responsible for the phenotypes in the mice is supported by a recently presented individual with PWS who has a paternally derived small genomic deletion comprising the entire *SNORD116* cluster and half of the *SNORD115* cluster. *SNORD116* expression was completely abolished (Sahoo T, del Gaudio D, German JR, Shinawi M, Peters SU, Person R, Garnica A, Cheung SW, Beaudet AL, Prader-Willi syndrome is caused by paternal deficiency for the HBII-85 C/D box snoRNA cluster. Presented at the annual meeting of The American Society of Human Genetics, October 26, 2007, San Diego, California. Available from http://www.ashg.org/genetics/ashg06s/index.shtml)

### Causes of postnatal growth retardation

Human infants with PWS have severe central hypotonia which makes them unable to suck effectively, and special feeding devices are often required. But despite adequate nutrition, they fail to thrive during infancy. Older children and adults with PWS have short stature in part due to growth hormone (GH) deficiency and respond well to GH treatment [29]–[31].

The *Snord116del* mouse model reproduces the severe neonatal growth retardation, but not the hypotonia. We were unable to document reduced milk intake, although stomach weights at P5 were reduced. Thus, it remains possible that a subtle difference in suckling behavior reduces milk intake, and cumulatively affects growth. Growth hormone secretion is not required during the first two weeks after birth, as shown by the normal growth in the first two weeks of the GH receptor knock-out mice [32]. Therefore, the neonatal growth retardation in *Snord116del* mice must be due to a defect other than lower GH secretion. The persistent growth retardation in puberty and adulthood in the deletion mice, however, is consistent with GH deficiency. No obvious difference in pituitary size or the number of GH producing cells was evident around the time of birth or in 4-week old mutants. Since GH secretion is pulsatile and the half-life of GH is very short (about 15–20 min), it can be difficult to demonstrate reduced circulating levels. To obtain a reliable assessment, we evaluated GH activity by measuring the mRNA and protein levels of IGF-I (insulin-like growth factor 1) and documented reduced *Igf1* mRNA levels in liver, as well as reduced IGF1 protein levels in the serum, at 4 and 8 weeks of age. Since *Igf1* transcription increases in response to GH binding, these results indicate an abnormality in GH release and/or signaling in the deletion mice. Our findings in the *Snord116del* mice are consistent with decreased circulating IGF-I in both GH-receptor (GeneID: 14600) and hypothalamic growth hormone-releasing hormone (GHRH) receptor (GeneID: 14602) deficient mice, that are approximately half and two-thirds the weight of their WT littermates, respectively [33]–[35] . Notably, circulating IGF-I levels in the GHRH (*lit/lit*) mice are in the range seen in our *Snord116del* mice (data not shown) suggesting a common final mechanism for the growth retardation related to available circulating IGF-I.

The literature on PWS has long suspected that the primary defect lies in hypothalamic dysfunction. While early autopsies of PWS brains revealed a reduction in the size of the paraventricular nucleus containing fewer oxytocin-preducing neurons and a 30% reduction in GHRH-producing neurons in the nucleus arcuatus of the hypothalamus [36], more recent studies of hypothalami from six adult obese PWS individuals did not confirm a reduction of GHRH-neurons [37]. Furthermore, GHRH and gonadotropin stimulation studies in humans demonstrated a lack of response in most PWS individuals, placing the site of the major defect into the pituitary rather than the hypothalamus [29]. Hypoplastic pituitaries have occasionally been reported in patients. In the *Snord116del* mice, we found normal pituitary development and structure, with normal representation of all five specific hormone-producing cell types. In comparison, in the *lit/lit* dwarf mice with a mutation in the GHRH gene (GeneID: 14601), the anterior pituitary is hypoplastic and GH-producing cells are reduced in number and are abnormally distributed [38].

Individuals with PWS have hypogonadism due to insufficient gonadotropin secretion, but their delayed puberty responds to GH treatment, indicating that it may be caused in part by deficient GH secretion [29]. The *Snord116del* females also have delayed puberty, as observed by delayed vaginal opening, in the absence of other signs of hypogonadism. We, therefore, propose that their delay in adolescent growth as well as in sexual maturation may be due to a defect in growth hormone release.

### Neurobehavioral features

The diagnostic criteria of PWS include developmental delay, cognitive impairment, and characteristic behavior problems such as temper tantrums, obsessive-compulsive behavior and stubbornness. In addition, a high pain threshold is noticed in many PWS individuals [2]. The neurobehavioral tests of *Snord116del* mice uncovered increased anxiety/fear in the elevated plus maze test, as well as impaired motor learning in the rotarod test. The elevated level of fear or anxiety may reflect higher stress level in the deletion mice, which is consistent with reports in PWS subjects. *Snord116del* mice responded as quickly to thermal pain as their WT littermates.

With respect to motor function, the mouse model differs from the human disorder. In PWS subjects, motor milestones are typically delayed by one to two years due to infantile central hypotonia. Although muscle tone improves with age, deficits in strength, coordination, balance, and motor planning may continue. Growth hormone treatment, by increasing muscle mass, can improve motor skills. In the *Snord116* deletion mouse model, we observed normal locomotor activity and excellent wire-hanging ability. Although initially the deletion mice performed similarly or even better than controls on the rotarod, they did not improve upon training, indicating a motor learning deficiency in the presence of good motor strength. Thus, we propose that in PWS subjects, the motor deficit may not solely be due to hypotonia, but an independent motor learning deficit may also be at play.

### Energy homeostasis regulation and food seeking behavior

What causes the hyperphagia and food-seeking behavior in PWS is unknown. At least three non-exclusive theories have been discussed. First, the hypothalamus, the center for energy homeostasis, may be defective. Second, neuro-imaging studies of PWS subjects indicated that the brain regions involved in reward and motivation, such as amygdala and medial prefrontal cortex, may be activated in the addiction-like food seeking behavior of individuals with PWS [39], [40]. Third, in studies of eating behavior, PWS individuals had longer meal times [41]–[43] and were insensitive to satiety signals [44]. We will now evaluate these hypotheses in the light of the results of our studies,

In *Snord116del* mice, the energy homeostasis regulation was normal under several testing conditions. First, the deletion mice were able to reduce their total food intake in response to higher caloric food and kept a more stable body weight than did WT controls. Second, the deletion mice were able to compensate for fasting with the same precision as the WT littermates, and that distinguishes *Snord116del* mice from other genetic models of obesity, such as *ob/ob* (leptin-null) mice, or those with hypothalamic neuronal ablation [22]. Third, the deletion mice were able to maintain their core body temperature in a 4°C environment, in contrast to many obese mouse mutants, such as *ob/ob*, *db/db* (null for leptin receptor), and *stat3*-/- mice, that quickly become hypothermic in the cold [24], [45]. Fourth, the *Snord116* deletion mice do not become obese even though the adults have increased daily food intake compared to WT. As we found no evidence of a deficiency in homeostasis regulation of food intake and energy expenditure, we propose that the pathophysiology of PWS does not involve a defect in energy homeostasis regulation, but rather, in behavioral aspects of overeating. Mutant mice, however, must have a way to maintain stable body weight despite their hyperphagia, a mechanism apparently lacking in humans with PWS.

Extensive studies on stress and feeding [46] indicate that increased stress may lead to increased cortisol levels and to increased feeding of palatable food. We considered the hypothesis that elevated stress might be the cause for hyperphagia in PWS, but found no evidence to support it. First, cortisol levels were reportedly normal in PWS children [47]. Second, the hyperphagia in PWS is not associated with seeking palatable food, but with a craving for any food items, and when restricted, even from trash. Third, during the first two days in the metabolic chamber, the WT mice reduced their food intake and lost body weight, which is a normal response to a stressful novel environment. The *Snord116del* mice, however, showed attenuated feeding inhibition, suggesting that their drive to feed overrides any fear and anxiety caused by the novel environment. In summary, in both human PWS subjects and our deletion mice, there is no evidence that increased levels of anxiety or stress is the cause for hyperphagia.

Based on eating studies in humans, it was proposed that PWS is a genetic model of starvation instead of food addiction [20]. Consistent with this proposal, PWS subjects do value food items more than other objects. In a recent functional MRI study, PWS individuals had higher activities of blood oxygen dependent response to pictures of food than to pictures of animals or tools. The signal was located in the medial pre-frontal region which is known to mediate reward [40]. These PWS subjects had normal insulin, glucose and triglyceride levels. Hence, the food seeking behavior is not caused by energy insufficiency, but rather by an abnormal response of the brain in the presence of sufficient fuel. Consistent with this notion, the *Snord116del* mice did not lose as much weight as the WT when their caloric intake was restricted. Thus, energy insufficiency is not likely to cause their hyperphagia.

Studies comparing food intake behavior of young PWS individuals and non-PWS obese controls showed that the PWS group spent more time eating, and ate more food [41]. Furthermore, PWS subjects, on average, ate three times more calories to attain a similar feeling of decreased hunger as did a lean control group [42], [48]. A detailed analysis of their eating behavior revealed that PWS children had a slower initial eating rate, but a much longer meal duration and non-decelerating eating curves [43]. Our *Snord116del* mouse model recapitulates the abnormal feeding behavior in human PWS. Their hyperphagia is associated with prolonged meal time, indicating a delay in meal termination that is most likely due to lack of sensing satiety.

Circulating neuropeptide hormones related to appetite regulation were measured in PWS subjects, and while leptin levels were similar to those in non-PWS obese subjects [49], plasma levels of ghrelin were elevated in adults [50]–[52]. Ghrelin is produced mostly in the stomach, and circulating levels are normally increased by fasting and reduced after feeding. Increased density of ghrelin-expressing cells was reported in PWS adult gastric tissues [53]. We found elevated circulating ghrelin in adult *Snord116del* mice which replicates the human data. It could be responsible for their hyperhagia, as in fed *Snord116del* animals, the ghrelin level was more than two-fold higher than in fed WT controls and resembled the fasting level in the WT mice.

It is puzzling that *Snord116del mice* eat more but retain a lean body composition. The metabolic profile of *Snord116del* mice showed decreased fat utilization, which is consistent with their elevated plasma ghrelin levels, as ghrelin administration in rodents leads to an increased respiratory quotient indicating less fat usage [54]. Ghrelin administration in normal rodents does not change energy expenditure, and, therefore, the ghrelin-induced decreased fat utilization leads to increased adiposity. In the *Snord116del* mice, however, energy expenditure is increased and body fat content is reduced. Thus, the metabolic profile of *Snord116del* mice does not recapitulate the effects of exogenous ghrelin administration.

Whether and how hyperghrelinemia causes hyperphagia in PWS individuals and *Snord116del* mice remains an open question. Since the gut hormone does not cross the blood-brain barrier in mice[55], any relevance of the hyperghrelinemia for the hypothalamic circuits that regulate feeding behavior is uncertain. In human PWS subjects, a short-term clinical trial of somatostatin treatment lowered plasma ghrelin level but failed to reduce appetite [56]. These results are difficult to interpret because somatostatin also suppresses the levels of anorexic peptides such as peptide YY. Future work crossing *Snord116del* mice with ghrelin-null mice and mice lacking the ghrelin receptor (also called growth hormone secretagogue receptor, GHS-R, GeneID: 208188) may define the role of ghrelin in the phenotype of *Snord116del* mice.

### Why is *Snord116* imprinted?

The function of imprinted genes in placental mammals is to regulate the growth of fetal and placental tissues [57]. Imprinting is thought to have evolved from a conflict between maternal and paternal genomes for the allocation of maternal resources. Paternally expressed genes promote growth (*Igf2, Peg1, Peg3, Ins1/2, Slc38a4*), and experimental knock-out of these genes restricts fetal and placental growth. Maternally expressed genes have the opposite effect [58]. While the main site of this intergenomic conflict is the placenta, we propose that the paradigm may extend to neonatal life. During the period before weaning, there is competition for maternal milk. Remarkably, the *Snord116del* mice were of normal size at birth and were not hypotonic, but failed to grow in the first three weeks of life, while they were dependent on maternal milk. As soon as they were weaned and eating on their own, their growth rate normalized. It is, therefore, possible that the primary function of the *Snord116* snoRNA is to promote postnatal growth during the pre-weaning period. PWS infants also fail to thrive during this time, even when nutrition is normalized via tube feeding. The “failure-to-thrive” condition may signal to the brain a state of starvation [20]. In response, the appetite regulatory circuits may become wired in such a way that the “lack-of-satiety” signal is permanently on and leads, in some unexplored way, to hyperghrelinemia and mealtime extension.

Not all imprinted genes can be functionally linked to the conflict hypothesis [59]. But not all genes in an imprinted domain need to be targets of this evolutionary selection; some could be bystanders, if imprinting is a genomic domain phenomenon. The functions of *SNRPN* and *NDN* do not fit well with the conflict hypothesis. Neither do the functions of the distal-less homeobox genes *DLX5* and *DLX6* whose imprinting status has recently been refuted [60]. Therefore, we propose that the intronic snoRNA cluster, rather than the SNRPN-protein coding part of the large transcript, or other genes such as *NDN*, is the target of selection for imprinted paternal expression of the 15q11.2 genomic domain.

### Conclusions

The *Snord116* mouse model provides a novel system to study the mechanism of hyperphagic behavior, and to test potential therapeutic interventions. Our studies of feeding behavior indicate that the meal size, as determined by the length of feeding bouts reflecting meal termination, is the key to the hyperphagic behavior, while energy homeostasis control is normal. This distinguishes the *Snord116del* mouse model from the genetic mouse models of obesity, including *ob/ob*, *db/db*, *Sim1* haploinsufficiency [61], *Mc4r* deletion [62] and others [63] that lack proper homeostasis control.

We propose that the primary mechanism for hyperphagia in PWS is not a defect in the energy homeostasis control, but in sensing satiety, and that higher cognitive regions of the brain, such as the medial prefrontal cortex, which is involved in interpreting incentive values of food, and those involved in planning complicated schemes to obtain food, may be changed secondary to the prolonged perception of hunger/lack of satiety. With respect to developing rational therapeutic interventions, we expect that drugs used for correcting addiction, which target the incentive control mechanisms in brain, will not be effective, and that research must focus on the sense of satiety required for meal termination. Since healthy lean individuals decelerated their rate of food intake within minutes from the start of a meal [43], meal termination is likely caused by a response to pre-absorptive cues. The *Snord116del* mice and PWS individuals may have a deficiency in sensing satiety signals pre-absorptively, such as vagal afferent nerve signals that transmit gastrointestinal distention, oral sensory input, and neuroendocrine peptides secreted directly by the gastrointestinal tract in response to distention.

From the genetic and mechanistic point of view, the most important next step is the identification of the target RNA(s) of *Snord116* and of the type of modification it catalyzes. Recognizing the involvement of a non-coding RNA in postnatal growth and appetite control represents the novel aspect of our work. The mouse model we created will allow future investigations into the mechanisms of early postnatal growth regulation, adult-onset hyperphagic behavior and metabolic adjustments that allow the mice to remain lean in the presence of hyperphagia with normal energy homeostasis,

## Materials and Methods

### Generation of targeting constructs

#### Upstream targeting construct

A 9 kb *Stu*I-*Bst*EII fragment was cloned from BAC RP23-371M8 (GenBank accession # AC026679, sequence antisense 125827-117210) and a 2 kb cassette containing a loxP site, and a PGK-Neo gene flanked by Frt1 sites (5′-GAAGTTCCTATTCTCTAGAAAGTATAGGAACTTC-3′) was subcloned into the *Pac*I site (AC026679, position 121632), which is ∼4 kb downstream of *Snord64*. A 1.2 kb *Bam*HI-*Eco*RI fragment of a PGK-diphtheria toxin cassette was purified from pBC-KS.DT7 (courtesy of Colin Stewart) and inserted downstream into the 9 kb genomic fragment. The finished construct was linearized before transfection.

#### Downstream targeting construct

A 7.4 kb *Eco*RV-*Mfe*I fragment was cloned from BAC RP23-309E15 (GenBank accession # AC026683, sequence 90280-98899). A cassette with puromycin resistance and thymidine kinase selective markers was constructed with flanking Frt5 sites (5′-GAAGTTCCTATTCTTCAAAAGGTATAGGAACTTC-3′) [64], [65], and a loxP site, and was subcloned into the *Bsr*DI site (AC026683. position 94582) which is ∼7 kb upstream of the first copy of *Snord115*. A 1.2 kb *Bam*HI-*Eco*RI fragment of a PGK-diphtheria toxin cassette was purified from pBC-KS.DT7 and cloned into the construct downstream of the genomic fragment. The finished construct was linearized before transfection.

### Generation of doubly-targeted (2-loxP) and deletion (1-loxP) ES cells

#### Generation of doubly-targeted (2-loxP) ES cells

BRUCE4 ES cells derived from C57BL/6 mice were a gift from Colin Stewart [66]. They were first transfected with the upstream targeting construct and placed in selection medium containing 200 µg/ml G418 (Invitrogen). DNA from resistant colonies was tested by Southern blot. First, the DNA was digested with *Nco*I and probed with probe UR, a 1.8 kb *Bst*XI fragment (AC026679, position 113455-115280) located downstream of the targeted region. The wildtype allele is represented by a 19.5 kb fragment, and the recombinant allele by a 12 kb fragment. The positive clones were subjected to a second Southern blot assay with probe UL, a PCR fragment (AC026679, position 128857–127788) located upstream of the targeted region. After *Nco*I digestion, the WT band is 19.5 kb and the recombinant band is 9.5 kb. Of 192 clones analyzed, only one clone (B41) passed both tests.

ES clone B41 was then transfected with the downstream targeting construct. Colonies resistant to selection in medium containing 1.5 µg/ml puromycin (Invitrogen) were tested by Southern blot assay for homologous recombination. First, the DNA from resistant clones were digested by *Pst*I and probed with probe DR (an *Mfe*I fragment of AC026683, position 98899-99822) located downstream of the targeted region. The WT allele generates a band of >16 kb and the recombinant allele a fragment of 6.5 kb. Next, the positive clones were subjected to a second Southern blot assay of *Xma*I digestion and hybridization to probe DL (AC026683, position 89169-89990), residing upstream of the targeted region. The WT *Xma*I fragment is 14 kb and the recombinant fragment is 8 kb. Of 96 colonies tested, 9 with good morphology passed both tests, and were labeled C1-C9.

To remove the Neo^r^ and Puro^r^-TK selective markers, the doubly targeted clone C3 was transfected with the FLP recombinase expression vector, pCAGGS-FLPe[67] and then grown in medium containing 0.2 µM FIAU (Moravek, CA) to select against TK activity. Cells were genotyped by a PCR assay (see below). Of 192 colonies tested, 43 were positive (E1–E43).

#### Generation of deletion (1-loxP) ES cells

Deletion (1-loxP) ES cells were generated by transfecting doubly targeted (2-loxP) ES cell clone C3 with a Cre recombinase expression vector pBS185 (Invitrogen) to induce recombination between the loxP sites that removed ∼150 kb sequence. The cells were selected in medium containing 0.2 µM FIAU (Moravek, CA). DNA from surviving colonies was tested by Southern blot using probe UL after *Nco*I digestion. The sizes of *Nco*I fragments representing the different alleles are: WT 19.5 kb, 2-loxP cells 9.5 kb, and 1-loxP cells 13.5 kb. The clones were also tested after *Stu*I digestion with probe DR. The fragment sizes are: WT>16 kb, 2-loxP cells 6.5kb, and 1-loxP cells 10 kb. A total of 14 positive clones (D1-D14) were obtained from 300 clones. They were all verified by the PCR genotyping assay.

### Genotyping by PCR

For genotyping 1-loxP ES cells and mice, we used primers PA (5′-TTTACGGTACATGACAGCACTCAAG, AC026679 position 121766-121742), PC (5′-AATCCCCAACCTACTTCAAACAGTC, AC026683 position 94254-94278) and PD (5′-TGGATCTCTCCTTGCTTGTTTTCTC, AC026683 position 94688-94664). The WT allele produces a PCR product of 435 bp with primers PC and PD, but no product with PA and PD since the distance between these primers is ∼150 kb. After CRE-induced deletion, primers PA and PD generate a product of 337 bp. PCR was performed using Taq polymerase (Promega) with 1.5 mM MgCl_2_, and the following cycling conditions: 95°C for 3 min; then 35 cycles of 95°C for 20 sec, annealing at 60°C for 20 sec, extension at 72°C for 90 sec; followed by a final extension at 72°C for 5 min, and cooling at 4°C.

For genotyping 2-loxP cells, we designed two sets of primers to test for the junctions in upstream and downstream targeted regions. In the upstream targeted region, PCR with primers PA and PB (5′-CATGCCAATGGTAAATCGTGG, AC026679 position 121388-121368) generates a 399 bp product of the WT allele; and no product of the doubly targeted allele in clones C1-C3 under the above cycling conditions, for the primers are separated by the 2 kb Neo^r^ marker. After FLP-induced deletion, a PCR product of 500 bp is generated. In the downstream targeted region, PCR with primers PC and PD produces a 435 bp product of the WT allele. In the doubly targeted allele in clones C1-C3, PC and PD are 4 kb apart and no PCR product is generated using the above conditions. After FLP-induced recombination, a PCR product of 510 bp is generated.

### Derivation of mice from targeted ES cells

We used albino C57BL/6J–Tyr^c-2J/J^ mice (The Jackson Laboratory, stock #000058) as the donor of host blastocysts. We obtained three chimeric mice from E3 (derived from C3) 2-loxP ES cells with 20–80% black coat color, and all of them transmitted the modified allele through the germ-line with more than 50% offspring derived from ES cells. The 2-loxP heterozygous mice were mated with homozygous C57BL/6-Tg(Zp3-cre)93Knw/J mice ((The Jackson laboratory, stock #003651). Doubly heterozygous (2-loxP/+; Zp3-Cre/+) females were mated with WT C57BL/6J males to obtained 1-loxP mice in the next generation. We also obtained two chimeric mice from injection of 1-loxP ES cells (clone D6) with 10% and 40% black coat color. Only the one with 40% chimeric color passed the deletion allele through the germ-line. Both 2-lox and 1-lox mice will be available from The Jackson Laboratory (the 2-loxP, the floxed conditional strain, C57BL/6-Snord116^tm1Uta^/J under stock # JR8118, and the 1-loxP, the targeted mutation, knock out, C57BL/6-Snord116^tm1.1Uta^/J under stock # JR8149.

### Quantitative RT-PCR Assays

RNA was isolated from brain and liver by using RNA STAT60 (Tel test) following manufacturer's instruction. RNA was treated with 0.5 U RNase-free DNase I (Ambion) for each µg of RNA at 37°C for 30 min, and was purified by phenol-chloroform extraction and precipitated with ethanol. Reverse transcription reactions were done using M-MLV reverse transcriptase (NEB) following manufacturer's instruction. For each 20 µl reaction, 2 µg of RNA was used. The RT reactions were diluted 1:25, and 2–5 µl of diluted reaction was used for quantitative PCR using the 2× polymerase mix (Applied Biosystem) in a 25 µl reaction, with 1 µl of 5 mM of each primers. We used a ABI 5700 real-time PCR machine. The primers for real-time quantitative PCR are: *Ndn*-F (5′-GCACCTGAGGCTGACCAATC) and *Ndn*-R (5′-CATGGGCATACGGTTGTTGAG); *Snrpn*-F (5′-TGCTACGTGGGGAGAACTTG) and *Snrpn*-R (5′-CCTGGGGAATAGGTACACCTG); *Snord107*-F (5′-GATGATATAGGACCTTGTCTG) and *Snord107*-R (5′-GATTTCAGTCACTGT AAGCTC); *Snord116*-F (5′-TGGATCTATGATGATTCCCAG) and *Snord116*-R (5′-TGGACCTCAGTTCCG ATGAG); *Snord115*-F (5′-GGGTCAATGATGACAACCCAATG) and *Snord115*-R (5′-GGGCCTCAGCGTA ATCCTATTG) [12]; *Ube3a*-F (5′-TGCACTGGTCCGGCTAGAG) and *Ube3a*-R (5′-TTCAAGTCTGCAGGATTTTCCA) [68]; *Gapdh*-F (5′-CTCCACTCACGGCAAATTCA) and *Gapdh*-R (5′-ATGGGCTTCCCGTTGATGA); *Igf1*-F (5′-GCTGGTGGATGCTCTTCAGTT) and *Igf1*-R (5′-GGTGCCCTCCGAATGCT) [69].

### Measurement of serum IGF-I levels by enzyme-linked immunosorbent assay (ELISA)

The procedures were carried out as described[70]. Monoclonal hamster anti-mouse IGF-I antibody (catalog # MAB 791), recombinant DNA-derived IGF-I (catalog #791) and biotinylated goat anti-mouse IGF-I antibody (catalog #BAF 791) were obtained from R&D Systems (Minneapolis, MN). Microtiter plates (96-well) were coated with purified monoclonal hamster anti-mouse IGF-I antibody at 0.5 µg/well in 100 µl of phosphate-buffered saline, incubated overnight at room temperature on a shaker, then washed three times with 300 µl/well wash buffer (PBS, 0.05% Tween-20), followed by 1 hr incubation with 300 µl/well blocking buffer (PBS, 5% Tween 20, 5% sucrose, 0.05% sodium azide) and three final washes with wash buffer. Standards were prepared by diluting recombinant DNA derived IGF-I in assay buffer (50 mM sodium phosphate, 150 mM NaCl, 0.1% Tween 20, 0.25% bovine serum albumin, pH 7.4) in concentrations ranging from 0 to 25 ng/ml.

Prior to IGF-I assay, 0.1 ml acid/ethanol reagent (12.5% 2 N HCl, 87.5% ethanol, v/v) was added to 0.025 ml of each serum sample. The mixture was incubated at room temperature for 30 min, microcentrifuged at 10,000 g for 10 min, and 0.05 ml supernatant was neutralized with 0.025 ml 1 M Tris base and diluted with IGF-I assay buffer to a final dilution of 40–100 fold. For the acid–ethanol extraction procedure, recovery of added mouse IGF-I was >90%. Standards, controls or prepared samples (50 µl/well) and 50 ng/well (50 µl/well) biotinylated goat anti-mouse IGF-I antibody were added to the prepared assay plates and incubated at room temperature for 2 hr on a shaker. Wells were then washed three times with wash buffer, followed by addition of 100 µl/well of Streptavidin–horseradish peroxidase (HRP) conjugate (Pierce Chemicals, Rockford, IL) and further incubated for 20 min at room temperature. After 4 washes with wash buffer, 100 µl/well of o-phenylenediamine hydrochloride (OPD) at 1 mg/ml in hydrogen peroxide Substrate (Pierce Chemicals) was added to each well and incubated for an additional 10–20 min. The reaction was stopped by the addition of 50 µl/well 2 N H_2_SO_4_ and absorbance was determined on a plate spectrophotometer (Molecular Designs, Sunnyvale, CA) at 490 nm.

### Neurobehavioral tests

All mice were housed in a facility with 12 hr light/dark cycles (lights on 7 am–7 pm). For all the neurobehavioral tests, only male mice were used. They were acclimated to the test room for more than 1 hr, and the tests were done between 8 am and 6 pm.

#### Open field test

The testing chamber is constructed of a plexiglass box of 48 cm×48 cm×26 cm, and the floor is divided equally into 16 squares. A center area of 12 cm×12 cm is labeled with red marker. Each mouse was given 5 min to explore the field and was video taped. The number of squares through which the mice traveled (3 paw entry), time spent in the inner square (2 paw entry), time spent in rearing and grooming were read from the video.

#### Elevated plus maze test

All mice were pre-handled at least once a day for 5 days before the tests. The maze was constructed with gray plexiglass. The four arms are 29 cm long and 5 cm wide. The open arms have 0.5 cm-wide edges that are 1 mm high to prevent mice from sliding off the arms. The closed arms have walls of 29 cm×29 cm with a dead end. The maze was elevated 50 cm above ground. The test mouse was placed at the center of the maze and given 5 min to explore. Activity was recorded on videotape for later analysis.

#### Y maze test

The Y maze was made from white plexiglass with three identical arms. Each arm is 30 cm×10 cm×10 cm. In the spontaneous alternation test, the test mouse was placed at the end of one arm and was given 7 min to explore. The starting arm for each mouse was randomized. The sequence of entry into each arm was recorded. All mice were pre-handled at least once a day for 5 days before the tests. In the novel arm exploration test, the maze was floored with soiled beddings, and one arm was blocked with a door. A mouse was placed in the end of one arm and was allowed to explore for 5 min. The bedding was stirred before each test. After 45 minutes, the third arm was opened, and the bedding was stirred and evenly distributed into all three arms. A mouse was placed in the same starting arm and was allowed 5 min to explore the three arms. Time spent in each arm and numbers of entries into each arm were recorded.

#### Wire-hanging test

A metal grid of 12 cm×16 cm, with bars spaced every 8.5 mm was hung 50 cm above the floor. Multiple layers of absorption pads were used as a cushion to catch the falling mouse. The test mouse was placed onto the grid and the grid was turned upside down. With the observation period of 150 sec, the latency to fall was recorded.

#### Rotarod test

The Columbus Economix (Columbus, Ohio) machine with eight tracks was used. All mice were pre-handled at least once a day for five days before the tests. The mice were given three trials of 90 sec on the rotarod at constant speed of 3 rpm to become familiar with the equipment. Afterwards, they were exposed to a 6 min trial with constant acceleration from 3 rpm to 45 rpm. Two trials with one hour interval were given on each day for 6 consecutive days. The time on top is defined as the time until the mouse either falls off the wheel or wraps around the wheel with passive rotation.

#### Hot plate test

A thermal metal block of 16 cm×9.5 cm was preheated to 52.5°C. The test mouse was placed on the metal surface and video taped. When it licked its hind paws or jumped, or the maximum time of 30 sec had elapsed, the mouse was removed from the hot plate. The video was analyzed at 4× slow-play mode.

### Feeding and energy homeostasis studies

#### Food intake measurement

Mice were individually housed for one week. They were switched to new cages with water and two pieces of food pellets. The food pellets were weighed within 1 hr after the onset of the light cycle, and 1 hr before the onset of the dark cycle. Regular rodent chow (Prolab 3000, 3.0 kcal/g; calories from carbohydrates: fat: protein = 60∶14∶26) was used if not specified. For high fat diet, we used HFD Research Diet D12451 (4.73 kcal/g; calories from carbohydrates: fat: protein = 35∶45∶20). For the fasting study, the mice were fasted for 24 hr with free access to water.

#### Core temperature in cold

The core temperature was measured with an anal thermometer (BAT-12, Ploytemp Instrument, NJ). The mice were placed into pre-cooled cages with food and water in a 4°C room, and their core temperature was measured every 30–60 min.

#### Body fat content measurement

The mice were anesthetized with 0.25% Avertin at a dose of 12 µl/g weight and were placed on the GE Lunar Piximus II densitometer using dual energy x-ray absorptiometry (DEXA) to measure body composition.

#### Glucose and insulin tolerance tests

For the Glucose Tolerance Test (GTT), the mice were fasted for 16 hr (6 pm–10 am), and then injected *i.p.* with 10% D-glucose solution at a dose of 1 g/kg weight. The blood glucose concentration was measured with a glucometer (Ascensia Elite, Bayer) at 0, 30, 60, 90 and 120 min. For the Insulin Tolerance Test (ITT), the mice were fasted for 4 hr (7 am–11 am), and then injected *i.p.* with recombinant insulin (Humulin, Eli Lily), and their blood glucose levels were measured as above. Different doses of insulin were used to obtain a maximum response without the mice passing out due to extreme hypoglycemia: males on chow, 1.6 U/kg; males on high fat diet, 2.0 U/kg; females on chow and on high fat diet, 1.0 U/kg.

#### Metabolic chamber study

Mice were placed into Oxymax System metabolic chambers (Columbus Instruments International Corporation, Columbus, OH) with water and food (rodent chow). Their locomotion, food and water intake, O_2_ consumption and CO_2_ emission were automatically monitored. RER (Respiratory Exchange Rate, also known as RQ) was calculated as VCO_2_/VO_2_.

#### Ghrelin measurement

Plasma was prepared from tail blood collected into ice-cold tubes with EDTA (final concentration 1mM) and protease inhibitor cocktail (Roche) between noon and 3 pm from 11 months old male mice with *ad libitum* access to food and water, or after 24 hr food restriction. Rat/mouse Ghrelin RIA kit (Phoenix Peptide, Belmont, CA) was used to determine ghrelin level.

### Statistical Analysis

Unless otherwise specified, all the data are expressed as mean±standard error of the mean. The *P* values were calculated using two-tailed *t*-test.

## Supporting Information

Figure S1 Immunohistochemical detection of pituitary hormone producing cells. On pituitary sections, all five pituitary hormone-producing cell types show normal abundance and appearance in Snord116del mice (Del) compared to WT littermates. a. Growth hormone labeling of somatotrophs in E18.5 pituitaries at 5× magnification. b. Hormone staining at 63× magnification: Thyroid stimulating hormone b subunit (TSHb) labels thyrotrophs, Pro-opiomelanocortin (POMC) labels corticotrophs in the anterior pituitary (AP) and melanotrophs in the intermediate lobe (indicated by the arrows), and luteinizing hormone b subunit (LHb) labels gonadotrophs in pituitaries of E18.5 embryos. PRL, prolactin labels lactotrophs in 4-wk old pituitaries.
(7.75 MB TIF)

Figure S2 Locomotion activity is not changed in mutant mice. The same group of mice as in Figures 6 A, B, C and D, were continuously monitored during 7 days in the metabolic chamber. No significant differences between Snord116del and WT mice were observed. Light and dark cycles are indicated on the horizontal axis. The vertical axis represents the locomotion activity per hour as measured by the numbers of infra-red beams the mice crossed in the metabolic chamber.
(0.61 MB EPS)
