# Supplementary figures and images for: SnoRNA Snord116 (Pwcr1/MBII-85) Deletion Causes Growth Deficiency and Hyperphagia in Mice
Source: PLoS One. 2008 Mar 5;3(3):e1709. doi: 10.1371/journal.pone.0001709 (PMC2248623; doi:10.1371/journal.pone.0001709)

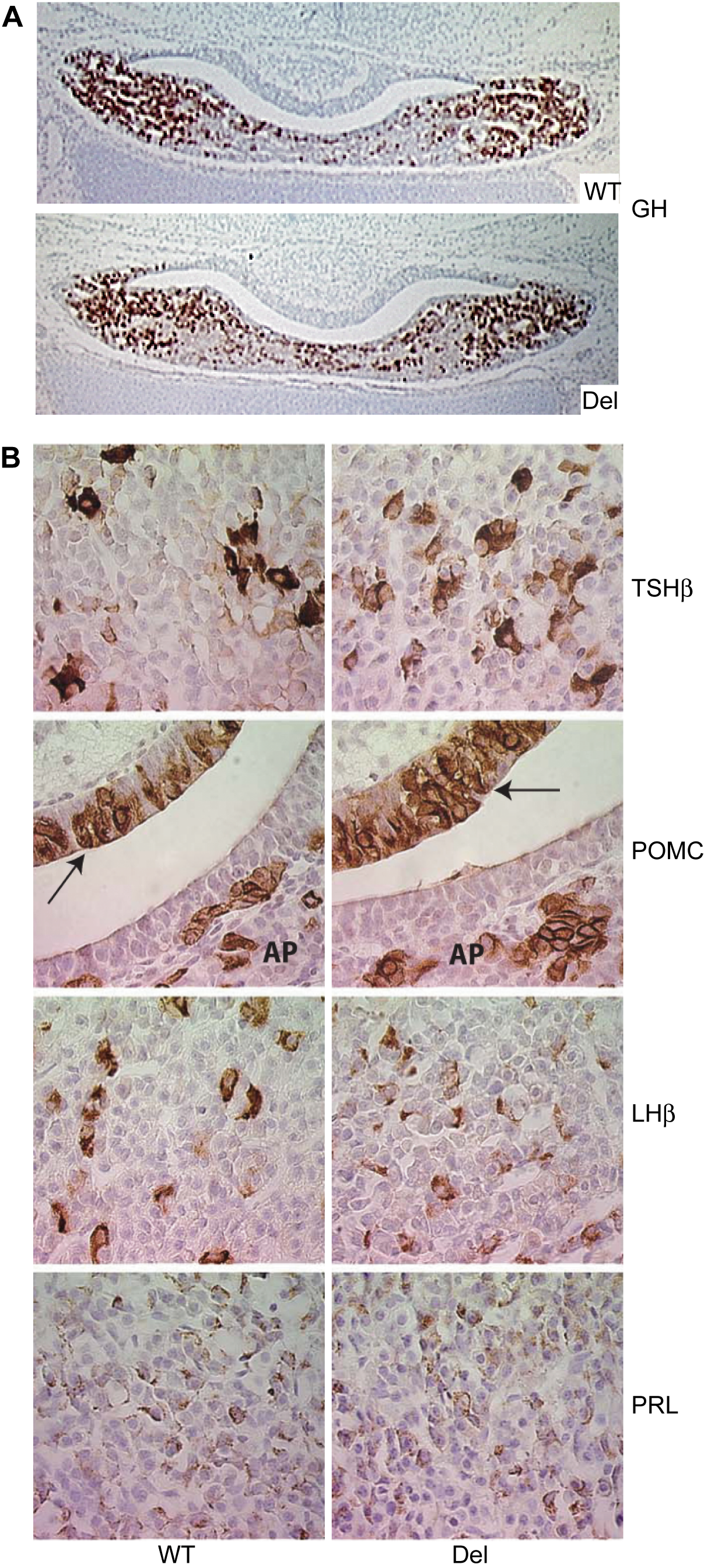

Supplement: Figure S1 — Immunohistochemical detection of pituitary hormone producing cells. On pituitary sections, all five pituitary hormone-producing cell types show normal abundance and appearance in Snord116del mice (Del) compared to WT littermates. a. Growth hormone labeling of somatotrophs in E18.5 pituitaries at 5× magnification. b. Hormone staining at 63× magnification: Thyroid stimulating hormone b subunit (TSHb) labels thyrotrophs, Pro-opiomelanocortin (POMC) labels corticotrophs in the anterior pituitary (AP) and melanotrophs in the intermediate lobe (indicated by the arrows), and luteinizing hormone b subunit (LHb) labels gonadotrophs in pituitaries of E18.5 embryos. PRL, prolactin labels lactotrophs in 4-wk old pituitaries. (7.75 MB TIF) [file pone.0001709.s001.tif]
